# Cross Deployment Networking and Systematic Performance Analysis of Underwater Wireless Sensor Networks

**DOI:** 10.3390/s17071619

**Published:** 2017-07-12

**Authors:** Zhengxian Wei, Min Song, Guisheng Yin, Hongbin Wang, Xuefei Ma, Houbing Song

**Affiliations:** 1 College of Computer Science and Technology, Harbin Engineering University, Harbin 150001, China; weizhengxian@sina.com (Z.W.); yinguisheng@hrbeu.edu.cn (G.Y.); wanghongbin@hrbeu.edu.cn (H.W.); 2 Science and Technology on Underwater Acoustic Autagonizing Laboratory, Systems Engineering Research Institute, Beijing 100094, China; 3 Information Technology Centre, Beijing Foreign Studies University, Beijing 100089, China; songmin@bfsu.edu.cn; 4 College of Underwater Acoustic Engineering, Harbin Engineering University, Harbin 150001, China; maxuefei@hrbeu.edu.cn; 5 Department of Electrical, Computer, Software, and Systems Engineering, Embry-Riddle Aeronautical University, Daytona Beach, FL 32114, USA

**Keywords:** UWSN, nodes deployment, networking, systematic performance

## Abstract

Underwater wireless sensor networks (UWSNs) have become a new hot research area. However, due to the work dynamics and harsh ocean environment, how to obtain an UWSN with the best systematic performance while deploying as few sensor nodes as possible and setting up self-adaptive networking is an urgent problem that needs to be solved. Consequently, sensor deployment, networking, and performance calculation of UWSNs are challenging issues, hence the study in this paper centers on this topic and three relevant methods and models are put forward. Firstly, the normal body-centered cubic lattice to cross body-centered cubic lattice (CBCL) has been improved, and a deployment process and topology generation method are built. Then most importantly, a cross deployment networking method (CDNM) for UWSNs suitable for the underwater environment is proposed. Furthermore, a systematic quar-performance calculation model (SQPCM) is proposed from an integrated perspective, in which the systematic performance of a UWSN includes coverage, connectivity, durability and rapid-reactivity. Besides, measurement models are established based on the relationship between systematic performance and influencing parameters. Finally, the influencing parameters are divided into three types, namely, constraint parameters, device performance and networking parameters. Based on these, a networking parameters adjustment method (NPAM) for optimized systematic performance of UWSNs has been presented. The simulation results demonstrate that the approach proposed in this paper is feasible and efficient in networking and performance calculation of UWSNs.

## 1. Introduction

Nearly 71% of the Earth’s surface is covered with water. As a result, the deep ocean is a vast and mostly unexplored habitat on our planet. Recently, however, there has been a growing interest in exploring and monitoring ocean environments either for scientific exploration, for commercial exploitation, or for navy military surveillance. Underwater Wireless Sensor Networks (UWSNs), as ideal systems for this type of extensive monitoring and exploration missions, have become a new hot research area [1,2]. UWSNs, employed in deep ocean exploration and underwater monitoring, are composed by three types of nodes: the master nodes, sensor nodes and mobile nodes, which together form a three dimensional (3D) network, popularly used in resource exploration, underwater monitoring and communication, etc. [3,4].

Some requirements need to be met in order to fulfill functions such as ocean exploration and underwater monitoring. Firstly, since the sensor nodes are distributed in a wide area range, a wide range of monitoring is required. Secondly, to collect and process data effectively, UWSNs are required to have high data transmission capability, including short delay, higher bandwidth and good QoS. Finally, UWSNs require longer lifetime and strong dynamic adaptation ability. Sensor nodes are deployed in a dynamic ocean environment, therefore the systematic performances of an UWSN such as coverage, connectivity and lifetime are impacted by the influence of parameters, including detection radius, communication radius and energy consumption rate. Hence, in designing the UWSN scheme, the systematic performance for the mission needs to be analyzed and evaluated first [5]. Moreover, UWSNs need to have the adaptive ability to adjust the communication radius, detection radius of sensors or routing, and the topology of the network to achieve longer lifetime when the environment and mission change.

Sensor nodes are deployed in a dynamic sea environment, and an UWSN is formed by networking to meet the needs of a specific mission. Therefore, these problems should be solved when the UWSN scheme is designed. On the one hand, an appropriate sensor node deployment strategy and networking mode are adopted to generate an UWSN which has superior system performance. On the other hand, through the evaluation of the system performance, the UWSN can optimally adjust sensor detection, communication radius and other parameters automatically to achieve longer lifetime or greater network coverage. Therefore, sensor node deployment, networking, and performance calculation of UWSN are challenging issues.

This study is conducted based on the following assumptions:

(1) The node types of an UWSN include master node, sensor node and mobile node. The master node is a vessel used for data processing. Sensor nodes are different kinds of stationary and submerged floating sensors deployed underwater. Mobile nodes are underwater unmanned vehicles, such as unmanned underwater vehicles (UUVs) and autonomous underwater vehicles (AUVs).
(2) In the beginning, sensor nodes are deployed randomly on the sea surface. When working, they can float on the ocean or be suspended in the ocean at certain depth. Sensor nodes are fixed in the water by anchoring. However, they can move in the vertical direction by changing their buoyancy, and slightly move back and forth horizontally with ocean current.
(3) The detection range of sensor nodes is a uniform ball-body [6]. They can only detect the targets precisely within the range, but cannot detect the targets outside of it. Sensor nodes have three states: active state, startup state and sleep state.
(4) The mobile nodes, mainly UUVs and AUVs carrying some sensors nodes, can redeploy and reconfigure their sensor nodes, and have the functions of resource exploration and environmental monitoring. Master nodes deploy sensor nodes and control the network.

Based on the above assumptions, this paper focused on the problems. In our initial study, CDNM and SQPCM of UWSN are proposed. More importantly a NPAM is newly presented. Different from other studies, in our approach the systematic performance of UWSNs, including coverage, connectivity, durability and rapid-reactivity have been calculated.

(1) We improve the normal body-centered cubic lattice (NBCL) [6] to cross body-centered cubic lattice (CBCL) for the deployment of UWSNs, and propose a Cross Deployment Networking Method (CDNM) for UWSNs suitable for the underwater environment. In the former NBCL, the body-centered cubic lattices structure is piled up layer by layer by regular ball-bodies. After improvement to the CBCL, the ball-bodies on the higher level are crossing stacked over the spaces between ball-bodies on the next layer, and the cubic space has been divided into hierarchies and monitored from top to bottom. Based on CBCL, a 3D UWSN deployment process and topology generation method are proposed. Then, based on CBCL and randomly deploy sensor nodes, a *K*-Connected *K*-Dominating Set (*K*-CDS) [7] is built as the virtual communication backbone, and a Cross Deployment Networking Method (CDNM) for UWSNs, suitable for the underwater environment, is further proposed.
(2) A Systematic Quar-Performance Calculation Model (SQPCM) is proposed. Considering the requirements of ocean exploration and underwater monitoring missions, we analyze the relationships between systematic network performance and the influencing parameters from an integrated view, and divide the systematic performance of UWSNs into four items, including coverage, connectivity, durability and rapid-reactivity. At the same time, we summarize the influencing parameters impacting the systematic performance items into three categories: constraint parameters, device performance parameters and networking parameters. Furthermore, the measurement models of coverage, connectivity, durability and rapid-reactivity performance are established. Then the network performance is evaluated and calculated systematically.
(3) In order to make UWSN have the adaptivity to adjust the network coverage, connectivity and effective run-time when the environment and mission change, a Networking Parameters Adjustment Method (NPAM) for optimized systematic performance of UWSNs is presented. The process of this method is composed of four parts, including state collection and detection, dynamic measurement model of an UWSN’s performance, optimal adjustment model of the UWSN and optimal method based on genetic algorithm. Through this method, an optimal combination of networking parameters can be achieved. As a result, it is possible for the network to have the maximum systematic performance in different missions and environment.
(4) Two simulation experiments have been carried out in this study, whose result demonstrates that the proposed approach is highly feasibility and efficient in UWSN networking and performance calculation.

The goal of our work is to obtain a network with the best system performance by using the CDNM and SQPCM and using as few sensor nodes as possible. At the same time, the dynamic optimization adjusted in the network is realized by the NPAM. The rest of the paper is structured as follows: Section 2 describes related works. Section 3 shows the CBCL for the sensor nodes deployment and the CDNM of UWSN. The systematic performance items analysis and the SQPCM of an UWSN are shown in Section 4. Section 5 describes the NPAM for optimized systematic performance of UWSNs. The simulations showing the result of our proposal are presented in Section 6. Finally, Section 7 gives the conclusions.

## 2. Related Works

With the increasing emphasis on effectively safeguarding national marine rights and interests, the upsurge in marine economic development and the significant progress in wireless sensor network technology, underwater sensor networks have become a new hot research area [1,2]. They are deployed to perform collaborative missions such as ocean organism data collection, ocean monitoring, offshore exploration, disaster prevention, assisted navigation and navy military surveillance. However, since the underwater acoustic communication channel has limited bandwidth capacity, the unpredictable underwater environment, the unique characteristics of underwater acoustic channels, the complex deployment spaces and the difficulties arising in translating the state-of-the-art theoretical UWSN designs into their underwater equivalent, there are many problems on deep ocean exploration sensors, UWSN architecture, routing protocols and energy management, intricate network design and deployment, underwater communications and networking technology. Numerous works [1,2,8,9,10] show that node deployment, networking and performance evaluation on UWSN are challenging issues and must be solved promptly.

Systematic performance analysis and calculation of UWSNs are becoming an important research topic. In [11], the coverage and connectivity issues for 3D UWSNs was investigated. Since node location can be random, redundant nodes have to be deployed to achieve 100% sensing coverage. Then a subset of the nodes can be dynamically chosen to remain active at a time to achieve sensing coverage based on their location at that time. One approach to achieve this goal in a distributed and scalable way is to partition the 3D network volume into virtual regions or cells, and to keep one node active in each cell. The results indicate that using cells created by truncated octahedral tessellation of 3D volume minimizes the number of active nodes. By adjusting the radius of each cell, this scheme can be used to achieve *k*-coverage [12]. The performance of these schemes for both 2D and 3D networks are analyzed and compared. When *k* = 1, *k*-coverage become 1-coverage. For 1-coverage, the 3D scheme is less efficient than the 2D scheme. The performance of 3D scheme improves significantly as compared to 2D scheme fork-coverage, for values of k is larger than 1.

Sensor deployment, networking and systematic performance calculations of wireless sensor networks must be based on network routing protocols. Garcia et al presented in [13] an overview about the main issues in underwater wireless ad-hoc networks, and ad-hoc routing protocols were discussed. In [14] a routing algorithm named Prolong-SEP (P-SEP) is presented, and in [15] a new group-based underwater wireless sensor network was proposed. In [16,17,18], the authors built a model named Routing and Multicast Tree based Geocasting (RMTG) for geocast region holes in underwater sensor networks and provided detailed analysis and performance evaluation for the model. Many other researchers have analyzed performance features such as connectivity, reliability and energy efficiency when studying specifically the routing protocol, network architecture and topology control for UWSNs [19,20,21,22].

Node deployment, networking and performance optimization are important research domains due to the changeable underwater environment, deployment at different depths, movement of nodes and so on. Devesh et al. pointed out in [23] that a sensor network has two competing objectives: (1) maximization of the network performance with respect to the probability of successful search for a specified upper bound on the probability of false alarms; (2) maximization of the network’s operable life. Then they presented an adaptive energy management policy that will optimally allocate the available energy between sensing and communication at each sensor node to maximize the network performance subject to specified constraints. In [24], Jiang et al. built a non-uniform deployment of nodes based on a clustering algorithm for underwater sensor networks. The algorithm is proposed because optimizing network connectivity rate and network lifetime is difficult for the existing non-uniform node deployment algorithms under the premise of improving the network coverage rate for the UWSN. Nie et al. have proposed an optimization method based on the genetic algorithm for surface gateway deployment, designed a novel transmission mechanism-simultaneous transmission, and completed two efficient routing algorithms that achieve minimal delay and payload balance among sensor nodes in [25]. From the related works, some conclusions can be drawn as follows:

(1) The correlation between the network systematic performance and the three kinds of parameters from an integrated perspective, that is, constraint parameters, device performance parameters as well as networking parameters, is an important research domain for UWSNs. In previous works, this topic has barely been discussed, with a few papers published.
(2) Currently there is a lack of systematic research on UWSN performance calculation and evaluation. Many researchers have analyzed the performance from the coverage, connectivity, reliability and energy efficiency perspectives separately to support their study of topics in such fields as network architecture, routing protocols and communication technology.
(3) There is no self-adaptive ability in networking, parameter adjustment and performance optimization of UWSNs. Many researchers have focused on network deployment, localization and self-adaptive energy management policy [13]. The networking parameters cannot be processed systematically after the UWSN is built.

## 3. Cross Deployment Networking Method

In this section we are going to improve the normal body-centered cubic lattice (NBCL) [6] to cross body-centered cubic lattice (CBCL) for the deployment of UWSNs, and propose a Cross Deployment Networking Method (CDNM) for UWSNs suitable for the underwater environment.

First, building an UWSN requires deploying the sensor nodes to the corresponding positions and organizing them into a network. Hence, the formation of an UWSN includes the network node deployment and networking. Node deployment is to place a certain number of sensor nodes in the designated area, to set up the cruising path of UUVs, and so forth. Networking mainly organizes the already deployed nodes and establishes the network topology, determines the routing policy and data transmission strategy of the network, and makes the network available to users. Therefore, the systematic performance of an UWSN depend not only on the physical performance of its nodes, but also on how these nodes are organized.

### 3.1. UWSN Deployment Process Based on Improved Body-Centered Cubic Lattice

**Figure 1:**
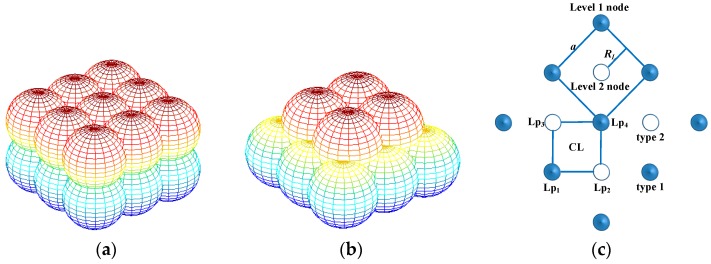
(**a**) NBCL, (**b**) CBCL and (**c**) Planar square lattice of NBCL.

The UWSN nodes need to be deployed in a certain environment, and they are influenced by the environment and the physical properties of the nodes themselves. The optimal ball-body coverage mode is a body-centered cubic lattice, whose normal coverage (NBCL) is shown in Figure 1a. In NBCL, the body-centered cubic lattices structure is piled up layer by layer by regular ball-bodies. If the boundary effect is ignored, this network topology needs less nodes for certain monitored spaces, but the ratio of energy consumption in the UWSN will become much larger, so we improve the NBCL to a Cross Body-centered Cubic Lattice (CBCL), shown as Figure 1b. In CBCL, the ball-bodies on the higher level are cross stacked over the spaces between ball-bodies on the next layer, and the cubic space is divided into hierarchies and monitors from the top down. The basic idea of CBCL generation is to utilize the vertical movement of sensor nodes to generate a similar body-centered cubic lattice from a planar square lattice. First, randomly distributed nodes on the plane are included into the Voronoi cell of different planar square lattices. Nodes in the same cell decide whether to enter the active state based on the states of other nodes in the cell. At the same time, the monitored space is divided in terms of the Voronoi cells of a body-centered cubic lattice. The moving depth of the sensor nodes is determined by the coverage scope of the body-centered cubic lattice. By moving in a vertical direction the nodes are deployed in the Voronoi cells of the body-centered cubic lattice hierarchically. With an adequate number of active nodes, the CBCL can form a 3D underwater monitoring network, which effectively reduces the energy consumption of the network without affecting the coverage scope.

If this CBCL structure is projected onto a plane it forms a planar square lattice CL shown in Figure 1c, and the planar square lattice of NBCL as CL. The Voronoi centre of each layer is distributed on the vertex of CL.

**Figure 2:**
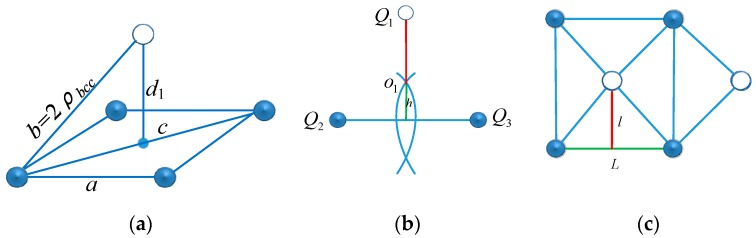
(**a**) Front view, (**b**) Common coverage area and (**c**) Vertical view.

We assume that the monitored space length is *x*, width is *y*, depth is *z*, detection radius of the sensor is *d_b_*, and the number of columns, lines and layers along the direction of *x*, *y*, *z* are *n*_1_, *n*_2_, *n*_3,_ respectively, and the distance between sensor nodes in the same layer is *a*. The position relationship of sensor nodes in NBCL when the monitored space is overcovered, is shown in Figure 2. In Figure 2a, the relationship between the *d_b_* and *a* is $\sqrt{2}d_{b}\leq a\leq 2d_{b}$. In Figure 2b, *h* represents the height of common coverage area among three sensor nodes, then *h* meets of $h=\sqrt{r^{2}-a^{2}/3}$, and the spacing of layers could be expressed with $H=Q_{1}O_{1}+h$.

When *d* = 2$d_{b}$, *h* = 0 and *H* = $\sqrt{2}d_{b}$. Figure 2c is the vertical view of the deployment of sensor nodes, and *l* and *L* represent the line distance and column distance of the nodes ranked according to the width and the length of the monitored space, respectively, then *l* = *a*/2 and *L* = *a*. Then the number of sensor nodes for the monitored space is *N*, hence, the formula (1) will be obtained:

(1) $$\begin{array}{c} \left(n_{1}-1\right)L\leq x,\left(n_{1}-1\right)L+L\geq x,\left(n_{1}-1\right)L+\frac{L}{2}\leq x\\ \left(n_{2}-1\right)l\geq y,\left(n_{3}-1\right)H+2h\geq z\\ N=n_{1}n_{2}n_{3}-⎣\frac{n_{2}}{2}⎦n_{3} \end{array}$$

In accordance with space geometry, the relationship between the coverage radius *R_bcc_* of the CBCL and coverage radius *R_CL_* of the planar square lattice CL can be computed. A unit of CBCL is shown in Figure 2a, in which the side length of the cubic body is *a*. Figure 2b shows the relationship between lattice packing radius *ρ_bcc_* and side length *a*, thus: *ρ_bcc_* = $\frac{\sqrt{3}a}{4}$ and *R_bcc_* = $\sqrt{\frac{5}{3}}\rho_{bcc}$, *R_bcc_* = $\frac{\sqrt{5}a}{4}$. Figure 1c shows a part of the planar square lattice CL, which is projected from the CBCL onto a plane. The coverage radius of the planar square lattice is the circumscribed circle radius of the Voronoi cell, thus *R_CL_* = *a*/2 and *R_CL_* = $\frac{2\sqrt{5}}{5}R_{bcc}$.

As all sensor nodes are deployed on the sea surface in the beginning, they can be seen as being on the same plane. The improved CBCL divides the nodes on the plane into different square cells according to the square lattice CL. We set the coverage radius of the square lattice CL to *R_CL_*_,_ then the coordinates of the reference point are (*x*_c_,*y*_c_), thus the coordinates of lattice point in CL are ($x_{c}+u\sqrt{2}R_{CL},y_{c}+v\sqrt{2}R_{CL}$), where *u* and *v* are integers. A square lattice can be uniformly represented by a binary set (*u*,*v*), thus (*u*,*v*) is used as the ID of the lattice point. The square cell ID of any node on the plane is that of the lattice point closest to the node.

In order to complete the 3D network node deployment, the sensor nodes must be sunk to different depths. The network is generated by planar nodes in accordance with the CBCL, so we need to distinguish the type of the square lattice point which the node belong to. According to Figure 1, the CL contains two types lattice point: type 1 and type 2. If the ID of Voronoi cell in type 1 and type 2 are respectively (*u*_1_,*v*_1_) and (*u*_2_,*v*_2_), we consider the following theorem:

**Theorem** **1.** *In square lattice CL, the sum of u_1_ and v_1_ in type 1 is W_1_ = u_1_ + v_1_, and the sum of u_2_ and v_2_ in type 2 is W_2_ = u_2_ + v_2_. One of W_1_ and W_2_ must always be odd, and the other must always be even.*

For example in Figure 1c, if the lattice point Lp_1_ is the type 1, then *W*_1_ = *u*_1_ + *v*_1_. If Lp_2_ belongs to type 2, then *W*_2_ = *u*_2_ + *v*_2_ = (*u*_1_ + 1) + *v*_1_, so if *W*_1_ is an odd integer, that the *W*_1_ must be an even integer.

**Figure 3:**
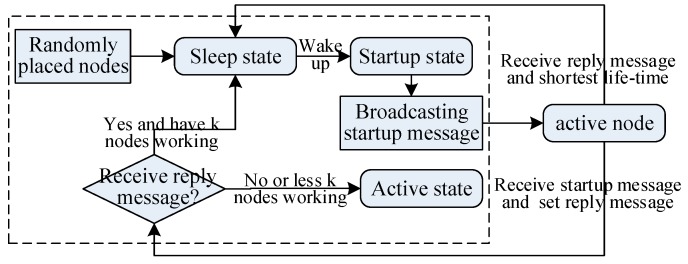
Network deployment processes and topology generation method.

Suppose the initial depth of the network monitoring 3D underwater space is *D*_0_, and its maximum depth is *D_m_*. According to the CBCL in Figure 1, the monitored space is stratified from top to bottom in the light of the plane of the CBCL. The distance between adjacent layers is called inter-laminar space, represented by *d*_l_, and as Figure 2a shows, *d*_l_ = $\frac{2\sqrt{5}}{5}R_{bcc}$, when the coverage radius of the *CBCL* is *R_bcc_*. Hence the depth of *i* layer in monitored space is *D*_0_ + *i*∙*d*_l_, *I* = 0, 1, ⋯, *n* − 1, where *n* = (*D_m_* − *D*_0_)/*d*_l_ + 1. Assume the square cell ID of a node on the plane is (*u*,*v*). Generally, the sensor nodes are deployed in the even layer when *u + v* is even; the sensor nodes are deployed in the odd layer when *u + v* is odd. Assume that sensor nodes are continually placed in the lattice points of the planar square CL in Figure 1, and they move down layer by layer according to the above method. In that way the network in the CBCL structure is generated. In practice, the positions of the sensor nodes on the plane are not overlapping. Some redundant nodes can be selected to generate a network in an approximate CBCL structure in the same way. As analyzed above, the network deployment process and topology generation method can be described as in Figure 3.

From Figure 3, the steps of the method are described as follows:

Step 1. Place the sensor node randomly on the ocean surface. After anchoring, it calculates the ID of the unit it belongs to according to its coordinates (obtained by GPS) and the pre-designated reference points, then enters into sleep state. The sleep time of nodes obeys the index distribution [7], and the probability density function is $f\left(t_{s}\right)=\lambda e^{-\lambda t_{s}}$, where *λ* is the startup speed; *t*_s_ is the sleep interval. The original value of parameter *λ* determines the interval in which the network wakes up enough nodes during the deployment phase.
Step 2. The node enters into startup state after awakening. The node broadcast startup messages within *R_p_* radius, which contains its own unit ID. To send the startup message to any nodes in the same cell, the *R_p_* should be set to satisfy the condition that *R_p_* ≥ 2*R_CL_*.
Step 3. For any active node which receives startup messages, if it belongs to the same unit as the starter node does, it sends a reply message which contains information of the layer where the sensor node is. Otherwise, it ignores the startup message.
Step 4. If the starter node does not receive any reply message within a certain interval, the sensor nodes will move to any layer of underwater monitored space from the surface and enter into active state. While the starter node receives the reply message, it first determines the number of active sensor nodes in this cell, and if there are *k* sensor nodes in active state in every layer, the sensor nodes go back into the sleep state. Otherwise, the sensor nodes move to a layer with less active nodes and enter active state.
Step 5. When the active nodes responds to the reply message from the neighbor node, they compare with the nodes with least work time in replying to the message only when there are *k* working nodes on the layer the node is in. It enters into the sleep state if its work time is shorter.

### 3.2. Cross Deployment Networking Method

Compared with radio communication, acoustic communication has low data transmission rates and high error rates. Therefore, in order to improve the reliability of data transmission and the parallel data transmission capabilities, we have built a *K*-Connected *K*-Dominating Set (*K*-CDS) as the virtual communication backbone, and proposed a Cross Deployment Networking Method (CDNM) of UWSNs suitable for the underwater environment. In CDNM, nodes on the communication backbone are responsible for data transmission. The users can query data by accessing the nodes on this communication backbone.

**Figure 4:**
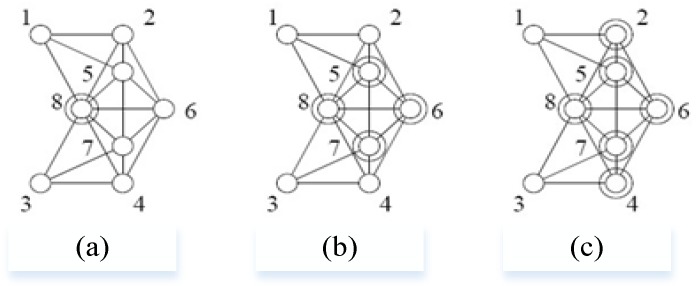
The examples of *K*-CDS: (**a**) *K* = 1; (**b**) *K* = 2; (**c**) *K* = 3.

Firstly, we introduce the concept of the *K-*CDS. A point set *V* is *K*-connected if and only if the network is still connected after any of the *K* − 1 points is taken out of this set. A point set *V*’ ⊆ *V* is *K*-dominated if and only if the nodes in set *V* are in dominating set *V*’ or there are *K* neighbor nodes in the dominating set. A point set *V*’ ⊆ *V* is *K*-connected *K*-dominated, if *V*’ is *K*-dominated, and the induced sub-graph of *V*’ is *K*-connected. As shown in Figure 4, *K* = 1, 2, 3 and 8 nodes form the *K*-CDS of the point set, in which points marked as concentric circles are selected into the *K*-CDS.

Because the nodes are deployed randomly, the neighbor relationships between nodes are also generated randomly. Therefore, during the *K*-CDS building process, we adopt the random method, namely, every node has a probability of *p_k_* of being a backbone node. The *p_k_* is a function that depends on *K* and the number of sensors. The algorithm of CDNM (Algorithm 1) is then described as follows:
**Algorithm 1:** CDNM1: Initial network nodes set
*NNS* = all node, virtual communication backbone nodes set *VCB_set* = Ø, no virtual communication backbone nodes set *NVCB_set* = Ø and setup the initial value of *p_k_*; Random select *N_i_* from *NNS* as initial node of virtual communication backbone; Put *N_i_* into *VCB_set*; 2: **For**(*j* == 0; *j* <= the node number of *NNS*; *j*++)3:   Random select *N_k_* from other nodes in *p_k_* probability4:  **If**(*N_k_* is not a member of *VCB_set* and *N_k_* + *VCB_set* meeting *K*-CDS)5:   Put *N_k_* into *VCB_set*;6:  **else**7:   Put *N_k_* into *NVCB_set*;8:  **end if**;9: **end for**;10: create routing protocol on OSPF;11: **end**.

When *p_k_* is appropriate, there is a high probability that the network can be built with *K*-CDS and CDNM. For example, in a network which includes 200 nodes, 2-CDS can be built with a probability of 98.2% when *p_k_* is 50%, 3-CDS can be built with a probability of 97.4% when *p_k_* is 60%. The advantage of this approach lies in its simplicity: it can build *K*-CDS without interaction with other nodes around it and it is especially suitable for environments with poor underwater communication conditions. After the *K*-CDS is completed, the network begins to generate the routing, namely, to build the routing table between *K*-CDS.

## 4. Systematic Performances Analysis of UWSNs

### 4.1. The Systematic Performance Items Analysis

UWSNs are composed of master nodes, sensor nodes and mobile nodes, performing functions such as ocean exploration and underwater monitoring. Firstly, UWSNs can be seen as an “extension tentacle” of underwater detection, which requires monitoring a wide area where the sensor nodes are distributed. Secondly, to collect and process data effectively, UWSNs are required to have high data transmission capability, including lower delay, higher bandwidth and good QoS. In addition, underwater monitoring needs a long time for information collection and processing, therefore the energy of the sensor nodes must be managed effectively for longer lifetime. Moreover, because of harsh underwater environment, sensor nodes may fail or recover frequently, so the network must have a high dynamic adaptability and fault tolerance. Therefore, from a systematic view, the systematic performance of an UWSN can be divided into the following items:

(1) *Coverage*. This is the effective monitoring scope as a network system, including the precision of detection within the monitored space. The stronger the coverage, the wider the network’s coverage, and any place within the range will have more nodes working simultaneously within the underwater space.
(2) *Connectivity*. This includes the degree of connection and data transmission capacity provided by the network. Stronger connectivity provides more end-to-end transmission paths and higher data transmission bandwidth and reliability.
(3) *Durability*. This is the run time duration of the network. Stronger durability ensures that the network can provide a longer exploration and monitoring service.
(4) *Rapid-reactivity*. This refers to the capacity to recover to normal network mode when a node is added to the network, fails in the network or the network mode is switched. Stronger rapid-reactivity represents a stronger ability to cope with dynamic changes when one node is added or exits, and when there is need to change the working mode.

The systematic performance of UWSNs depends not only on the physical performance parameters of the nodes, but also on the constraints of the underwater monitoring mission and the network structure. Hence, the relationship between systematic performance items and the influencing factors of UWSNs should be established. From the above analysis, the factors influencing the performance of UWSNs can be divided into three categories: constraint parameters, device performance parameters and networking parameters. The expectation performance of UWSN in the monitoring mission determines the constraint parameters, which include the UUVs’ and sensor nodes’ contribution rate to systematic performance items, the adjustment coefficients for different systematic performance items, and so forth. Constraint parameters only relate to the evaluation standards, but not on the actual devices and their physical performance. Therefore, once the evaluation standards are made certain, the constraint parameters are determined. The velocity of mobile nodes, the detected radius and precision of sensor nodes, etc., are the device performance parameters. The communication radii of sensor nodes and the number of working nodes will affect the network topology, and then lead to the change of network routers and data transmission capability, and such parameters are called networking parameters. The parameters and their labels are listed in Table 1.

In Table 1, *p_b_* mainly includes the communication energy consumption and the detected energy consumption. The *k_b_* represents the number of different active nodes recorded by a node over a certain interval after its neighbor nodes receive the message and is regarded as the number of its neighbor nodes. This node takes the minimum of neighbor nodes as *k_b_*. In UWSN, *c_b_* is influenced by *d_b_*and *n_b_*, *k_b_*is influenced by *r_b_* and *n_b_*, *p_b_* is determined by *r_b_* and *d_b_*. Within the definite monitored space, when the number of nodes in the network can at least cover the entire region, then *n_b_* is influenced by *d_b_*. Therefore, the key factors are *r_b_* and *d_b_*. The increase of the detection radius can lead to a corresponding decrease of the number of nodes, and then a decrease of the connectivity *k_b_* and an increase of the energy consumption. The bigger the communication radius of the node is, the more energy is consumed and the louder the background noise will be. However, the benefit lies in the connectivity which should be improved while under the same node deployment density.

### 4.2. Systematic Quar-Performance Calculation Model of UWSN

The systematic performance is influenced by constraint parameters, device performance parameters and networking parameters. In this section, we will propose a Systematic Quar-Performance Calculation Model (SQPCM) of UWSN, and in this SQPCM, the systematic performances items measurement formulas based on these parameters are built.

#### 4.2.1. Measurement Formula of Coverage

Coverage is denoted as *Θ*, which is determined by the coverage scope and the detection capacity within the coverage scope. Assuming the underwater detection scope of the sensor node is a standard ball-body, then the coverage volume of each node is about $\frac{4}{3}\cdot \pi \cdot d_{b}^{3}$, for all sensor nodes, and the coverage scope of the network is about $\frac{n_{b}\cdot \frac{4}{3}\cdot \pi \cdot d_{b}^{3}}{c_{b}}$. Because an UUV can move to a specified location in underwater space, its contribution rate to the network coverage scope is related to its location: when an UUV locates inside an UWSN, its contribution is zero; when an UUV locates at the edge of an UWSN, there is a direct proportional relationship between its contribution and its detection scope. The coverage scope of the UWSN can be measured by Equation (2):

(2) $$\frac{n_{b}\cdot \pi \cdot d_{b}^{3}}{c_{b}}+\theta \cdot n_{u}\cdot d_{u}^{3}$$

In this equation *θ* represents coefficients. *θ* > 0 stands for the average contribution rate of the UUV to the coverage scope, and it is dominated by the proportion of the vehicle cruise route inside and outside the UWSN.

Detection performance of the network is dominated mainly by the average degree of coverage and the precision of node detection. A wider average degree of coverage and higher detection precision guarantees stronger detection performance of the network. The average degree of coverage is mainly influenced by the number of sensor nodes. In addition, UUVs can detect the nearby area while cruising, so detection performance can be represented by Equation (3):

(3) $$\left[1-{\left(1-h_{b}\right)}^{c_{b}}\right]+\varphi \cdot h_{u}$$

In this equation, *ϕ* represents coefficients, and *ϕ* > 0 indicates the UUV’s average contribution rate to detection performance, which depends on the proportion inside and outside the UWSN when the UUV is cruising. In summary, the coverage of network can be measured by Equation (4):

(4) $$\Theta =\alpha \cdot \frac{n_{b}\cdot \pi \cdot d_{b}^{3}}{c_{b}}\cdot \left[1-{\left(1-h_{b}\right)}^{c_{b}}\right]+\beta \cdot n_{u}\cdot d_{u}^{3}\cdot h_{u}$$

In the equation, *α*, *β* > 0 represents the relative contribution coefficient between the sensor network and the UUV.

#### 4.2.2. Measurement Formula of Connectivity

Connectivity is denoted as *Φ*, which is determined by the quality of a single link and degree of connection of the network.

Network connectivity is not only impacted by the degree of connection between sensor nodes, but also can be enhanced when an UUV is used as the mobile communication node. In addition, these mobile nodes can be seen as working sensor nodes inside the network. Therefore, the degree of connection is measured by Equation (5):

(5) $$k_{b}+\mu \cdot v_{u}\cdot n_{u}$$

In this equation, *μ* > 0 represents the contribution that the UUV makes to the network, and the value of this parameter is determined based on the cruising path of the UUV.

Connection links including underwater and overwater links. The underwater links, whose quality is affected mainly by sensor node underwater channel transmission capacity, are mainly established through acoustic channels, and there is inverse proportional relationship between bandwidth and distance. Moreover, in a short distance, an UUV can transmit data to the master node by cable with high bandwidth and reliability. Overwater communication is achieved mainly through radio, and its bandwidth and distance depend on the sending and receiving power. Therefore, the link quality can be measured by Equation (6):

(6) $$\omega \cdot \left[\left(1-\eta \right)\cdot b_{b}+b_{u}\right]+\left(1-\omega \right)\cdot b_{b}^{\prime }$$

In this equation, *ω*, *η* are coefficients, 0 < *η* < 1 represents the error rate of the underwater acoustic channel; 0 < *ω* < 1 represents a ratio of the underwater communication to the overwater communication.

Finally, the data must be sent to the master node, so the data transmission capacity of this master node may affect the connectivity performance of the network. When the transmission bandwidth of the master node is large enough, the transmission bandwidth of the network can be fully used. When the velocity of the master node is fast enough, even partial data cannot be transmitted through the sensor nodes, then the master node can move over and collect the data. Therefore, Equation (7) is adopted to show the effect of the master node on the network data transmission:

(7) $$I=\frac{v_{s}\cdot n_{s}}{\max\left(B-b_{s},1\right)}$$

In the equation, *B* is the largest underwater bandwidth required for data transmission.

In summary, connectivity can be measured by Equation (8):

(8) $$\Phi =\alpha \cdot k_{b}\cdot \left[I\cdot \omega \cdot \left(1-\eta \right)\cdot b_{b}+\left(1-\omega \right)\cdot b_{b}^{\prime }\right]+\beta \cdot I\cdot v_{u}\cdot n_{u}\cdot b_{u}$$

In this equation, *α*, *β* are adjustment indexes.

#### 4.2.3. Measurement Formula of Durability

Durability is denoted as *Ψ*, which is determined by the lifetime of the nodes in an UWSN. The lifetime of the network is the interval from the moment that the network starts working to the moment that *m* nodes shut down due to energy exhaustion, which means that the lifetime of the network is determined by the first *m* nodes whose energy is exhausted. The *m* is a parameter that is determined by network redundancy. The lifetime of a single node is determined by the total energy and working energy consumption speed. Furthermore, on its cruising path, an UUV can substitute for some nearby sensor nodes to carry on the data transmission function, therefore, the lifetime of those sensor nodes will be extended. The durability of the network can be measured by Equation (9):

(9) $$\Psi =\alpha \cdot \left[\underset{m}{\mathrm{Min}}\left(\frac{e_{b}}{p_{b}}\right)+\beta \cdot \frac{e_{u}}{p_{u}}\right]$$

In the equation, Min*_m_* returns the *m*-smallest element in the set; *β* > 0 represents the UUV's contribution to the lifetime of nodes, which is associated with the cruising path of the UUV. *α* is the capability adjustment coefficient. *p_u_* is the UUV's energy consumption, including primarily the moving energy consumption, communication energy consumption and detection energy consumption, among which the moving energy consumption is the main part; *p_b_* is the energy consumption of the sensor nodes, including primarily the communication energy consumption and detection energy consumption. The communication energy consumption has a direct proportional relationship to the cube of the communication radius; the detection energy consumption has a direct proportional relationship to the cube of the detection radius, shown by Equation (10):

(10) $$p_{b}=\alpha \cdot r_{b}^{3}+\beta \cdot d_{b}^{3}$$

In the equation, *α*, *β* are coefficients, and *α* is associated with the communication rate of the current nodes. The energy consumption of the radio communication process is not considered in Equation (10), mainly because that the energy consumption of the radio communication is minimal compared with that of the acoustic communication, and the data communication by the electromagnetic waves is also relatively less.

#### 4.2.4. Measurement Formula of Rapid-Reactivity

Rapid-reactivity is denoted as *Ω*, which is determined by the network delay and network redundancy. Whether due to the network initialization or abnormal nodes adding or exiting, the delay is determined mainly by the communication delay and waiting mechanism. In UWSNs, the maximum delay occurs in the process of communication between nodes of the network diameter ends, where *V* is the acoustic speed in water, and *L* is the network diameter. Therefore, the maximum delay of the network diameter direction is $\tau =\frac{L}{V}$. Assume that the error rate is *η* for each jump through acoustic communication, then the success rate of communication in network diameter direction is $\varphi ={\left(1-\eta \right)}^{\frac{L}{r_{b}}}$.

In the specific network protocol, assume that the standard waiting time for retransmission is *s* times of the round-trip time in network diameter direction, and the number of retransmission waiting periods is *l*, then the average waiting time for the network diameter direction is represented by Equation (11):

(11) $$t=\overset{\infty }{\underset{k=0}{\sum }}{\left(1-\varphi \right)}^{k}\varphi \cdot (\tau +k\cdot \tau \cdot s\cdot l)=\tau \cdot \left(1+\frac{s\cdot l}{\varphi }\right)$$

If we set *k*_b_ as the network redundancy, rapid-reactivity can be measured by Equation (12).

(12) $$\Omega =\frac{\alpha \cdot k_{b}\cdot b_{b}}{\frac{L}{V}\cdot \left(1+\frac{s\cdot l}{\varphi }\right)}$$

In the equation, $\varphi ={\left(1-\eta \right)}^{\frac{L}{r_{b}}}$, *α* > 0 is the network redundancy influence factor.

### 4.3. Map Relationship Between Systematic Performance Items and Influence Parameters

**Figure 5:**
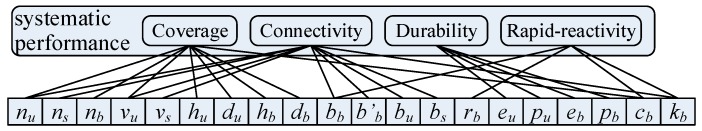
Map relationship between performances and parameters.

From the four measurement formulas above, we can see there is a certain relationship among those four items, and they are determined by basic performance parameters. The coverage and connectivity are impacted by the number of UUVs and sensor nodes, while durability and rapid-reactivity are impacted by the number and coverage scope of sensor nodes, and rapid-reactivity and connectivity are impacted by the communication range diameter and degree of connection. Therefore, a map relationship between the performance items and the parameters is established, as shown in Figure 5.

## 5. Networking Parameter Adjustment Method for Optimized Systematic Performance

The underwater environment and monitoring mission will vary as time changes. UWSNs need to have adaptive ability of optimization and adjustment with the variation of the environment and mission. In this section, a Networking Parameters Adjustment Method (NPAM) for optimized systematic performance of UWSNs is presented.

There are three categories of parameters influencing an UWSN’s performance: constraint parameters, device performance parameters and networking parameters. Table 2 presents the categories of various performance items and parameters. In practice, it is necessary to achieve an optimal combination of these parameters so that the network can achieve the best effect for different missions or in different environments, with minimal cost.

**Figure 6:**
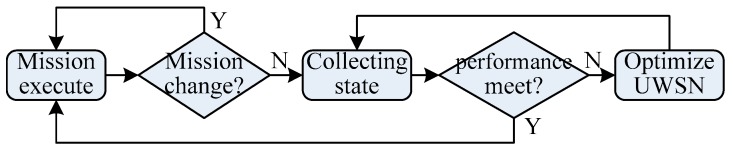
Process of UWSN optimization.

When the underwater environment changes or the current monitoring mission varies, the network needs to be adjusted optimally. In order to ensure optimization and adjustment are done reliably and in a timely way, there is a need to have a control center in the network. It is reasonable that the master node be regarded as the control center, so that all data should be collected at the master node, and then be processed. It dominates the network to be adaptive for optimal adjustment. The process of network optimization is shown in Figure 6.

### 5.1. UWSN State Collection

According to the analysis above, in the optimal adjustment of the network, the concerned parameters include *n_b_*, *p_b_*, *r_b_*, *k_b_*, *d_b_* and *c_b_*, in which *n_b_* can be obtained by periodically broadcasting messages in the network, namely, it can be obtained by counting the number of times a transmitted key heartbeat message is received and replied to by the active nodes in an interval. The counting waiting time is set as twice the longest delay in the network. The UWSN nodes read *e_bi_* (*t*), the remaining energy at *t* in every Δ*t* period, and compute the average energy at *t* through the expression *p_bi_*(*t*) = (*e_bi_*(*t*) − *e_bi_*(*t −* Δ*t*))/Δ*t.*

When the nodes reply to the heartbeat message, the control node collects the average power of all nodes and *p_b_* can be obtained by computing the mean of all average powers. The *r_b_* and *d_b_* can be self-controlled by these nodes, so they can be obtained in time and all of this information can be sent back to the control node. During nodes’ data transmission in the way of broadcast, the neighbor nodes of the control node receive the message and record it, and then reply to this message to the original node. The number of nodes is recorded in a period as the number of neighbor nodes. When the node replies to the heartbeat message, the minimal number of neighbor nodes that the control node captures is considered to be *k_b_*. In accordance with the network topology, each node collects the state information of all the other nodes in this lattice cell, records the number of active nodes, and transmits the information to the control node. The minimal number of neighbor nodes in all lattices is regarded as the current average coverage degree *c_b_* in this network.

### 5.2. Systematic Performance Calculation of UWSN’s

The control node collects the corresponding state information periodically in the network. Quantified values of the coverage, connectivity, durability and rapid-reactivity performance can be computed through Equations (2) to (12). These quantified values are compared with the current mission requirements. If each performance item of this network satisfies the mission demands, the control node is not changed and adjusts the network topology and works continuously. If one or more performance item cannot meet the demands, the network should be optimized and adjusted.

**Figure 7:**
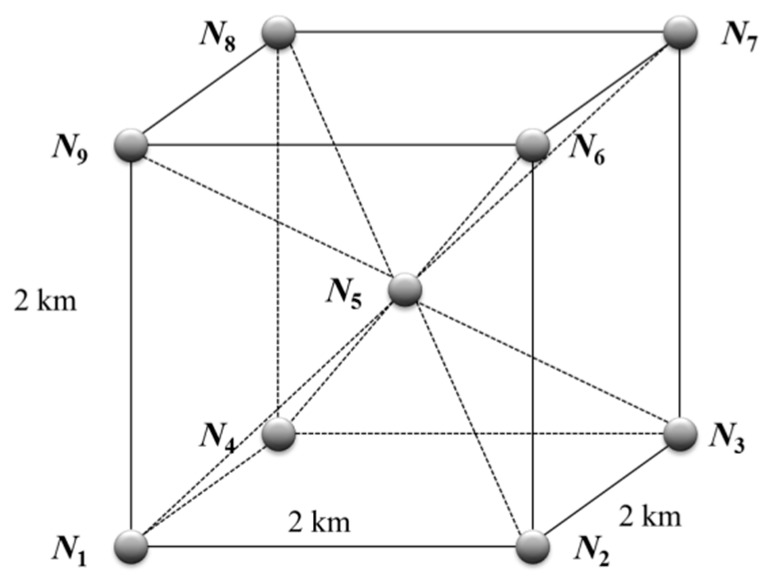
The ideal underwater sensor network.

In order to provide an intuitive evaluation for the quantified values of each systematic performance item, this paper assumes an ideal UWSN as shown in Figure 7. Suppose there are nine sensor nodes distributed within 2 × 2 × 2 km^3^, and *N*_1_–*N*_5_ can only communicate with each other underwater, and *N*_6_–*N*_9_ can do it both underwater and overwater. Other performance features of the sensor nodes are *r_b_* = 2 km, *b_b_* = 5 kbps, *b’_b_* = 5 Mbps, *d_b_* = 1 km, *h_b_* = 100%, *k_b_* = 4, *c_b_* = 1, and *e_b_* = 10^6^ J, for *e_b_*, the lifetime of sensor nodes is 10^7^ s (about four months) in normal working state. In addition to the sensor nodes, the network contains a master node and a UUV, and their performance features are *v_s_* = 5 m/s, *v_u_* = 2 m/s, *b_u_* = 20 kbps, *d_u_* = 1 km, *h_u_* = 100% and the lifetime of the UUV is 7 × 10^4^ s (about 20 h). Assume that the velocity of acoustic channel is 1500 m/s, and the bit error rate of transmission is *η* = 0.1. Suppose the value of each systematic performance item in the network is 1. According to the abovementioned assumption, the values of the constraint parameters can be determined.

When we substitute the above parameter values into Equation (4), then Equation (13) will be obtained:

(13) $$9\times 10^{9}\cdot \pi \cdot \alpha +10^{9}\cdot \beta =1$$

Since the sensor nodes play a dominant role in the coverage, their significance is supposed to be 0.9 and that of the UUV is supposed to be 0.1. Then *α* and *β* can be described by Equation (14):

(14) $$\alpha =\frac{1}{10^{9}\cdot \pi },\;\beta =10^{-9}$$

Then Equation (4) can be rewritten as Equation (15).

(15) $$\Theta =\frac{n_{b}\cdot d_{b}^{3}}{10^{9}\cdot c_{b}}\cdot \left[1-{\left(1-h_{b}\right)}^{c_{b}}\right]+10^{-9}\cdot n_{u}\cdot d_{u}^{3}\cdot h_{u}$$

In a similar way, we set *B* = 40 kbps, then *I* = 0.25. In order to decrease the radio communication, the data transmitted over water that is rare but important is just for some urgent control message. Assume the proportion of data transmitted under water is *ω* = 0.9999. Putting these values in Equation (8), we have 6.5·α+10·β=1. The two terms in this equation denote the sensor nodes’ and UUV’s contribution to connectivity. Setting *α* = 0.14, *β* = 0.009, their contribution ratios are 0.91 and 0.09. Generally, Equation (8) can be rewritten as Equation (16):

(16) $$\Phi =1.4\times 10^{-5}\cdot k_{b}\cdot \left[8999.1\cdot I\cdot b_{b}+b_{b}^{\prime }\right]+0.009\cdot I\cdot v_{u}\cdot n_{u}\cdot b_{u}$$

For durability, suppose the energy consumption speed of each node is the same. The coefficients *α* and *β* are constant and we have $8\times 10^{9}\cdot \alpha +10^{9}\cdot \beta =0.1$. Also suppose the ratio of communication energy consumption to the total energy consumption is 80%, take *α* = 10^−11^, *β* = 2 × 10^−11^, then $p_{b}$ can be described by Equation (17):

(17) $$p_{b}=10^{-11}\cdot r_{b}^{3}+2\times 10^{-11}\cdot d_{b}^{3}$$

Putting these parameters in Equation (9), we have $\alpha \cdot \left(10^{7}+7\times 10^{4}\cdot \beta \right)=1$, where *β* is the contribution coefficient of the UUV to the durability of the network. Assuming *β* = 20, the contribution proportion of the UUV to the network is about 14%, so Equation (9) can be rewritten as Equation (18):

(18) $$\Psi =\frac{1}{1.14\times 10^{7}}\cdot \left(\frac{e_{b}}{p_{b}}+20\cdot \frac{e_{u}}{p_{u}}\right)$$

For the rapid-reactivity, suppose the waiting time of overtime retransmission is twice the round time transmitted along the diameter, and the waiting times of retransmission is *l* = 3, then we will have 0.9382·α=1, that is, *α* = 1.0658. Hence, Equation (12) can be rewritten as Equation (19):

(19) $$\Omega =\frac{1.0658\cdot k_{b}\cdot b_{b}}{\frac{L}{V}\cdot \left(1+\frac{s\cdot l}{\varphi }\right)}$$

It should be pointed out that this case is not unique. It can be redefined according to practical monitoring mission demands and the environment, and the corresponding parameters can be recalculated.

### 5.3. The Process of the Networking Parameters Adjustment Method

If the control node’s evaluation result of the current network shows that the values of systematic performance items on network are not suitable for the current mission, the network should be optimally adjusted to achieve the requirement. According to the analysis above, the networking parameters include *k_b_*, *d_b_*, *n_b_*, *c_b_*, *r_b_*, and *p_b_*. Since the location of most sensor nodes are determined, within the given monitored space, the covering degree *c_b_* of network is affected by the detection radius *d_b_* and the number of nodes *n_b_*; and the connection degree *k_b_* is affected by the communication radius *r_b_* and the number of nodes *n_b_*. In CBCL, their relationships are shown by Equation (20):

(20) $$\begin{array}{c} 1\leq c_{b}<\frac{\frac{4}{3}\pi \cdot d_{b}^{3}\cdot n_{b}}{X\cdot Y\cdot Z},\;c_{b}\leq k_{b}<\frac{4}{3}\pi \cdot {\left(\frac{r_{b}}{d_{b}}\right)}^{3},\\ ⎡\frac{X}{d_{b}}⎤\cdot ⎡\frac{Y}{d_{b}}⎤\cdot ⎡\frac{Z}{d_{b}}⎤+⎡\frac{X}{d_{b}}+1⎤\cdot ⎡\frac{Y}{d_{b}}+1⎤\cdot ⎡\frac{Z}{d_{b}}+1⎤\leq n_{b} \end{array}$$

In the equation, *X*, *Y*, and *Z* together determine the volume of the monitored space. The maximum network coverage does not exceed the detection range of all nodes; the maximum connectivity of the network is not larger than the number of all nodes within the communication range. In addition, in order to increase the network connectivity, the proportion of communication radius and that of detection radius of the network should be increased. The increase of connectivity has a step trend and the step function is denoted as *f* (*m*). From Equation (17), it can be observed that the energy consumption *p_b_* of a node is determined by *r_b_* and *d_b_*. Therefore, systematic performance items of the network can be optimally adjusted by modifying the communication radius, detection radius of nodes and the number of active nodes so as to make them different from the initial values $\Theta_{0}$, $\Phi_{0}$, $\Psi_{0}$ and $\Omega_{0}$.

Since there are many scenarios satisfying the parameter values, a better solution should be chosen according to the mission demands. For example, in order to extend the lifetime of a network, it is necessary to take the minimum of the energy consumption speed of network as the optimal objective, that is, the minimum of $n_{b}^{2}\cdot p_{b}$. This objective in fact constrains the maximum of *n_b_*, *r_b_*, *d_b_* to extend the network lifetime. In this scenario, the adjustment model is written as Equation (21):

(21) $$\begin{array}{c} Minimize:n_{b}^{2}\cdot p_{b}\\ \Theta_{0}-\frac{n_{b}\cdot d_{b}^{3}}{10^{9}\cdot c_{b}}\cdot \left[1-{\left(1-h_{b}\right)}^{c_{b}}\right]-10^{-9}\cdot n_{u}\cdot d_{u}^{3}\cdot h_{u}\leq 0\\ \Phi_{0}-1.4\times 10^{-5}\cdot k_{b}\cdot \left[8999.1\cdot I\cdot b_{b}+b_{b}^{\prime }\right]-0.009\cdot I\cdot v_{u}\cdot n_{u}\cdot b_{u}\leq 0\\ \Psi_{0}-\frac{1}{1.14\times 10^{7}}\cdot \left(\frac{e_{b}}{p_{b}}+20\cdot \frac{e_{u}}{p_{u}}\right)\leq 0\\ s.t.\;\;\;\Omega_{0}-\frac{1.0658\cdot k_{b}\cdot b_{b}}{\frac{L}{V}\cdot \left(1+\frac{s\cdot l}{{\left(1-\eta \right)}^{L/d_{b}}}\right)}\leq 0\\ 10^{-11}\cdot r_{b}^{0}+2\times 10^{-11}\cdot d_{b}^{3}-p_{b}\leq 0\\ 1\leq c_{b}<\frac{\frac{4}{3}\pi \cdot d_{b}^{3}\cdot n_{b}}{X\cdot Y\cdot Z},\;c_{b}\leq k_{b}<\frac{4}{3}\pi \cdot {\left(\frac{r_{b}}{d_{b}}\right)}^{3},\\ 0<f_{\left(m\right)}\cdot d_{b}\leq r_{b}\\ ⎡\frac{X}{d_{b}}⎤\cdot ⎡\frac{Y}{d_{b}}⎤\cdot ⎡\frac{Z}{d_{b}}⎤+⎡\frac{X}{d_{b}}+1⎤\cdot ⎡\frac{Y}{d_{b}}+1⎤\cdot ⎡\frac{Z}{d_{b}}+1⎤\leq n_{b} \end{array}$$

In this equation, $\Theta_{0}$, $\Phi_{0}$, $\Psi_{0}$ and $\Omega_{0}$ denote the initial expectation values of the coverage, connectivity, durability, and rapid-reactivity. *k_b_*, *d_b_*, *n_b_*, *c_b_*, *r_b_*, and *p_b_* are the optimum parameters which can be obtained by solving the equation. If the equation has not been solved, the current network does not meet the mission demands and needs to be optimized and adjusted, or more sensor nodes are required to fulfill the mission. However, if this equation can be solved, the responding parameters are sent to each node and their node values can be adjusted so as to satisfy the mission needs. After the first round of optimization, the control node needs to evaluate the systematic performance of network again. After the third round of optimization, if the demands of the mission is still not satisfied, the state of current node does not fit the demands of the adjusted parameters. The reasons for not achieving theoretical optimum parameters include the following: firstly, in practical deployment, the sensor nodes of a network do not always have an accurate body-centered cubic structure and the calculation result may have deviations. Secondly, the results of the evaluation of connectivity, coverage and so on are not so accurate, and the parameter values need to be calculated according to the practical deployment circumstances. Lastly, there may be no deployment schedule which is in exact accordance with the optimal result. For instance, perhaps a node must be placed at a certain location in theory, but no node can reach that position, hence the optimization will fail, and a new round of optimization is required.

In order to adjust the parameters for optimized systematic performance of UWSNs, this paper employs a genetic algorithm to solve the non-linear optimal problem with constraint conditions.

### 5.4. Optimization Method Based on a Genetic Algorithm

A genetic algorithm simulates the bio-evolution process of genetic choice and natural selection and combines the rule of survival of the fittest and the mechanism of stochastic information exchange of part of the chromosomes in a group. It is an effective parallel searching method that can achieve global optimal solutions. Aiming at meeting the optimal demand proposed in this thesis, the optimization method based on genetic algorithm is presented as follows:

(1) Individual encoding and initial population

Encode: Real encoding is employed. We set six parameters *k_b_*, *d_b_*, *n_b_*, *c_b_*, *r_b_*, and *p_b_* as *x*_1_, *x*_2_, *x*_3_, *x*_4_, *x*_5_, and *x*_6_. We regard *x*_1_, *x*_2_, *x*_3_, *x*_4_, *x*_5_, and *x*_6_ as six pieces of a gene which construct a chromosome, and as an individual.

(2) Evaluation of fitness

(a) The fitness

The fitness function is the key to evaluate individual and determines the evolution strategy. The optimal objective function: $f\left(x\right)=x_{1}^{2}\cdot x_{6}$, so the fitness function is shown as Equation (22):

(22) $$Fit\left(f\left(x\right)\right)=\frac{1}{1+c+f\left(x\right)},c\geq 0,c-f\left(x\right)\geq 0$$

In the equation, *Fit* denotes the fitness function, and *c* is a constant.

(b) Constraint conditions

The constraint conditions process is achieved by independently defining a measure for these constraint conditions or a measure characterizing the degree of individual violation of the constraint conditions. Then, a better individual is selected by comparing and competing the fitness value with the unfitness values of the individuals. The degree of violation of the constraint conditions is defined by Equation (23):

(23) $$obj\left(x\right)=\overset{l}{\underset{j=0}{\sum }}\max\left(0,g_{i}\left(x\right)\right),1\ll j\ll l$$

In the equation, *obj*(*x*) is the function of individual violating the constraint conditions; $g_{i}\left(x\right)$ is the constraint conditions in formula (21); *l* is the number of constraint conditions.

(3) Selection

Set a threshold *ε* > 0 as a given degree of violating the constraint conditions, and the following rules are used to compare two individuals *A* and *B*:

(a) When the two individuals *A* and *B* are feasible, the individual with a larger fitness value is regarded as optimum by comparing the values *f*(*A*) and *f*(*B*).
(b) When the two individual *A* and *B* are unfeasible, the individual with a smaller unfitness value is regarded as optimum by comparing the values *obj*(*A*) and *obj*(*B*).
(c) When individual *A* is feasible but *B* is not, if *obj*(*B*) ≤ *ε*, the individual with a larger fitness value is regarded as optimum by comparing the values *f*(*A*) and *f*(*B*). Otherwise, *A* is regarded as a better individual.

Evidently, the bigger the values of *ε*, the higher the proportion of unfeasible solutions. In order to keep the proportion of unfeasible solutions in the population at a given rational level *p* > 0, an adjusted strategy for *ε* is shown as indicated by Equation (24):

(24) $$\{\begin{array}{c} \varepsilon^{\prime }=1.2\cdot \varepsilon ,\;p_{i}\leq p,1\leq i\leq K\\ \varepsilon^{\prime }=0.8\cdot \varepsilon ,\;p_{i}>p,1\leq i\leq K\\ \varepsilon^{\prime }=\varepsilon ,\mathrm{others} \end{array}$$

For a given positive integer, from the first generation of produced feasible solutions in the population, we compute the proportion *p_i_* (1 ≤ *I* ≤ *K*) of unfeasible solutions in the population of every generation in *K* generations and rectify *ε* as *ε*′. Through the above selection operation, better individuals can be identified. Meanwhile, a proper and better unfeasible solution can be reserved in the new population.

(4) Crossover

Based on the above process of constraint conditions, an arithmetic crossover operation of feasible solutions and unfeasible solutions is employed, that is, two new individuals are produced by linear combination. The operation of arithmetic crossover is shown in Equation (25):

(25) $$\begin{array}{c} x_{A}^{t+1}=ax_{B}^{t}+\left(1+a\right)x_{A}^{t}\\ x_{B}^{t+1}=ax_{A}^{t}+\left(1-a\right)x_{B}^{t} \end{array}$$

In the equation, *a* is a random number in [0, l], $x_{A}^{t}$ denotes the *t*-th generation gene of individual *A*, and $x_{B}^{t}$ denotes the *t*-th generation gene of individual *B*.

(5) Mutation

The unequal mutation shown by Equation (26) is employed:

(26) $$x_{k}=\{\begin{array}{c} x_{k}+\left(u_{k}-x_{k}\right)\cdot r{\left(1-\frac{t}{T}\right)}^{b},random\left(0,1\right)=0\\ x_{k}-\left(u_{k}-l_{k}\right)\cdot r{\left(1-\frac{t}{T}\right)}^{b},random\left(0,1\right)=1 \end{array}$$

where *r* is a random number in [0, 1]; *T* is the greatest number of heredity generations; *t* is the number of current heredity generations; *b* is the unequality parameter; *x_k_* is the *k*-th gene of an individual, and *l_k_* < *x_k_* < *u_k_*, *random*(0,1) is a random function producing a random number between 0 and 1.

The key reason that exact optimal result can be obtained quickly through this algorithm lies in the core parameters set of this algorithm, shown as follows: popsize = 200; the cross probability *P_c_* = 0.7; mutation probability *P_m_* = 0.1; the proportion of unfeasible solutions in population *p* = 0.2, *ε* = 0.1, the value of *ε* is adjusted as every *K* = 5.When the *T* = 4000 and the variation of average fitness is less than 0.01, the algorithm is terminated.

## 6. Simulation Experiment

We have run simulation experiment to investigate the feasibility and efficiency of our approach. Based on STK (3D display) and MATLAB (system performance calculation), we developed a simulation software for deployment, networking and system performance evaluation of UWSNs, which supports the random deployment and uniform deployment of sensors. The simulation parameters are set as follows: in a 12 × 12 × 4 km^3^ of monitored space where the sensor nodes are deployed, in the beginning, every sensor node is randomly deployed on the surface of the water, it can carry out underwater and overwater communication, where the maximum underwater communication radius is *r*_b_ = 4 km and maximum *d_b_* = 2 km, the communication bandwidth is *b_b_* = 10 kbps, and other parameters are *b’_b_* = 5 Mbps, *h_b_* = 100%, *e_b_* = 10^3^ kJ. Besides the sensor nodes, the network includes a master node whose parameters are *v_s_* = 5 m/s and *b_s_* = 40 kbps. The network also includes an UUV, whose parameters are *v_u_* = 2 m/s, *b_s_* = 20 kbps, *d_u_* = 2 km, *h_u_* = 100% and its lifetime is 7 × 10^4^ s (about 20 h) under normal working condition. At the same time, we assume that the velocity of the acoustic channel is 1500 m/s and the required maximum underwater bandwidth is *B* = 60 kbps.

In the simulation experiment, when the sensors were deployed, the UWSN is generated according to CDNM, the master node and UUV are randomly deployed in the monitored space, and the detection accuracy is set at 1. In practice, when the detected precision is less than 1, we need more sensors to increase the probability of detecting the target. We assumed there were enough sensors and the monitored space was overcovered. In the network, it is supposed that a target randomly entered the monitored space, the closest sensor detected the target and transmitted its information (at 28 bytes per second) to the master node through the CDNM routing.

### 6.1. Simulation Experiment on Networking and Systematic Performance Calculation of UWSNs

**Figure 8:**
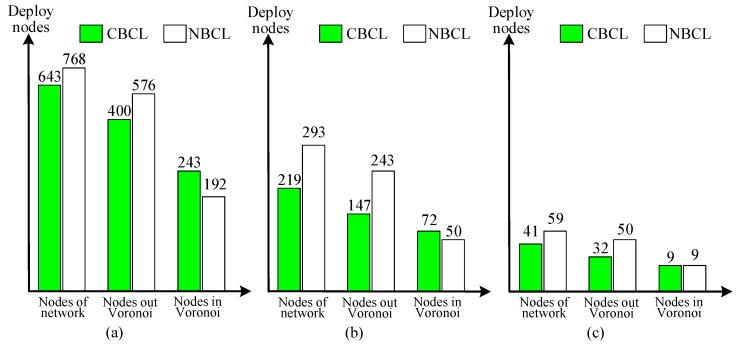
The nodes number on our approach and NBCL: (**a**) *d_b_* = 0.67; (**b**) *d_b_* = 1; (**c**) *d_b_* = 2.

Firstly, the different parameters that influence the systematic performance items are tested. The complete coverage of the network is crucial in every underwater monitoring mission. The nodes are deployed on CBCL and NBCL, and the CDNM is used to generate the network. It can work out the minimum number of sensor nodes, number of sensor nodes out of the Voronoi cells and the number of sensor nodes in the Voronoi cells where the detection radii of the nodes are 0.67 km, 1 km, 2 km, as shown in Figure 8.

Form Figure 8, for the coverage of network, it can be seen that the detection radii of the nodes are 0.67 km, 1 km, 2 km, so the number of nodes in our approach, compared with NBCL for the same monitored space, is separately decreased by 16.3%, 25.3% and 30.5%. At the same time, it is observed that the number of the required nodes decreases when the detection radius of the node increases. This is because that the number of required nodes is inversely proportional to the cube of the detection radius due to the 3D deployment of nodes underwater.

**Figure 9:**
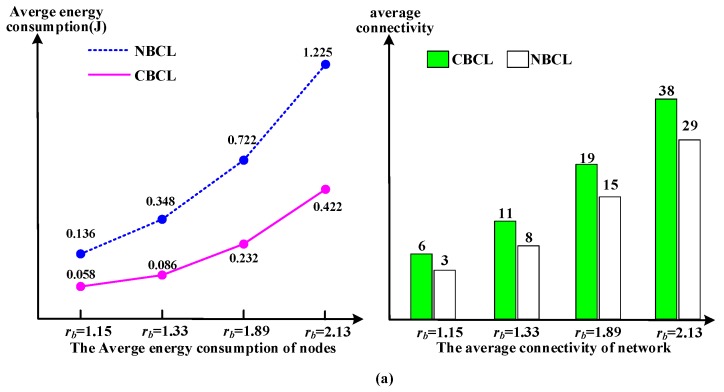

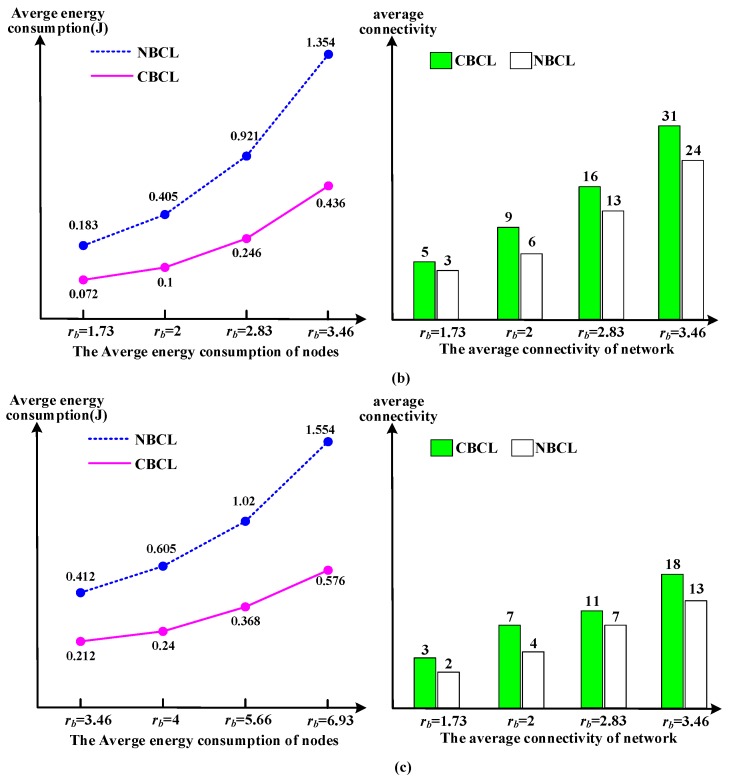
The energy consumption and connectivity: (**a**) *d_b_* = 0.67; (**b**) *d_b_* = 1; (**c**) *d_b_* = 2.

In addition, both the detection and communication radius of the nodes determine the energy state of nodes. In light of the different detection radii, when the communication radii of the detection radii are changed at different times, different average energy consumption of nodes can be obtained. Moreover, the network connectivity is associated with the number, detection radii and communication radii of sensors, as shown in Figure 9.

From Figure 9, for the detection and communication radii of sensor nodes, the average energy consumption and the connectivity in our approach is better than that in the NBCL. For the connectivity, it is observed that the bigger the communication radius of a sensor node is, the greater the corresponding connectivity will become.

In CBCL, from the right part of Figure 9a, when the detection radius is *d_b_* = 0.67, the communication radii are *r_b_* = 1.15, *r_b_* = 1.33, *r_b_* = 1.89 and *r_b_* = 2.13, respectively, and the corresponding average connectivities of the network are 6, 11, 19 and 38. On the contrary, the bigger the detection radius of a sensor node is, the less the corresponding connectivity will become, and from the right part of Figure 9b,c, the detection radii *d_b_* = 1 and *d_b_* = 2, that the corresponding average connectivities are 31 and 3 when the communication radius was *r_b_* = 3.46.

Durability is determined by the first *m* nodes whose energy is exhausted. The lifetime of a sensor is impacted by the total energy and energy consumption rate. For the average energy consumption of sensors, it is observed that the bigger the communication radius of a sensor node is, the greater the corresponding energy consumption will become. In CBCL, from the left part of Figure 9a, when *d_b_* = 0.67, *r_b_* = 1.15, *r_b_* = 1.33, *r_b_* = 1.89 and *r_b_* = 2.13, that the corresponding average energy consumption of the sensor node is 0.183, 0.405, 0.722 and 1.225. The same relationship occurs between the detected radius and node energy consumption.

Without considering wake-up of nodes joining the network, we assume that the lifetime of an UWSN is the interval from when the network starts working to when 20% of the active sensor nodes shut down due to energy exhaustion. For Figure 9c, the UWSN lifetime is shown in Table 3. 

**Figure 10:**
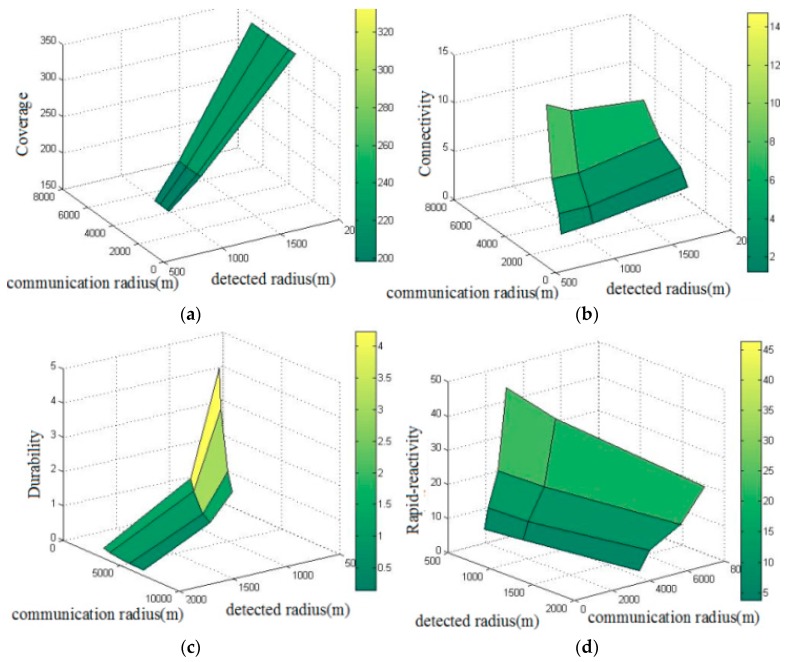
Relationship performances and parameters: (**a**) Coverage; (**b**) Connectivity; (**c**) Durability; (**d**) Rapid-reactivity.

From Table 3 we can see that the more active sensor nodes in the network, the longer lifetime the UWSN enjoys. According to Equations (6) to (10), for the detection radius and communication radius in Figure 9b, a relationship between the value of the systematic performance items and communication radius and detection radius can be observed, as shown in Figure 10.

As shown in Figure 10a, the network coverage increases as the detection radius of the node increases. This is because the network coverage is only associated with the detection radius of the nodes, but not with the number and communication radius of the nodes when it reaches 1-coverage.

Figure 10b shows that the connectivity of network increases as the communication radius increases. This is because when the communication radius of a node increases, that of the nodes connected with it will increase accordingly. Meanwhile, the connectivity diminishes as the detection radius increases due to the influence of the number of nodes. Under the condition of 1-coverage, the bigger the detection radius of the nodes is, the fewer the number of nodes needed. If the communication radius remains unchanged, reducing the number of nodes can lead to a decrease of the number of neighboring nodes, so the connectivity of network will be weakened.

Figure 10c shows that the durability of the network diminishes as the communication radius and detection radius of nodes increase. This is because the bigger the communication radius and detection radius of nodes are, the greater the node energy consumption rate will become, which leads to a reduction of the network’s lifetime.

Figure 10d shows that the rapid-reactivity of a network increases as the communication radius increases and it diminishes as the detection radius of the nodes increases. The phenomenon is related to connectivity. When the communication radius of a node increases, the connectivity of the network also increases. This can improve rapid-reactivity. When the number of nodes diminishes, the connectivity of the network diminishes too, and the number of nodes that can be used as redundant nodes also decreases, which leads to a weakening of rapid-reactivity. 

In conclusion, our approach proves that CBCL and CDNM are better than NBCL, and at the same time, compared to some previous works ([19] (2011), [20] (2013), [21] (2014), [22] (2015), [26] (2016) and [27] (2017)), the SQPCM can be employed to evaluate the systematic performance of a network on coverage, connectivity durability and rapid-reactivity, as shown in Table 4.

### 6.2. Simulation Experiment on NPAM of UWSNs

In order to verify the Networking Parameters Adjustment Method (NPAM) for optimized systematic performance of UWSNs, and referencing the parameter values in Figure 9b, this part considers setting the systematic performance values of the optimal goal as follows: $\Theta_{0}=200$, $\Phi_{0}=3$, $\Psi_{0}=1$, $\Omega_{0}=10$, so it realizes the optimal operation for Equation (21). All initial values of variables, the intermediate results and the optimized of parameters are shown in Table 5.

In the first round, we set $f\left(m\right)=\sqrt{3}$, namely, the smallest ratio of the communication radius, to be the detection radius. The optimization results are shown in line 2 of Table 5. Because of the estimation method, *k_b_* is higher than the actual value. Then the nonlinear optimization function makes *c_b_*, *k_b_* be decimals. Actual values of these parameters can be obtained in the subsequent evaluation. After the first round, the network sets up the corresponding parameters according to the optimized adjustment scheme proposed in this paper, and we then evaluate and calculate the performance items according to the evaluation method. It is found that the actual value of the connection degree is 5, and the actual values of the performance items are $\Theta_{1}=227$, $\Phi_{1}=2.0148$, $\Psi_{1}=1.1087$, $\Omega_{1}=6.0261$, which cannot meet the requirements, so the optimization adjustment must continue.

**Figure 11:**
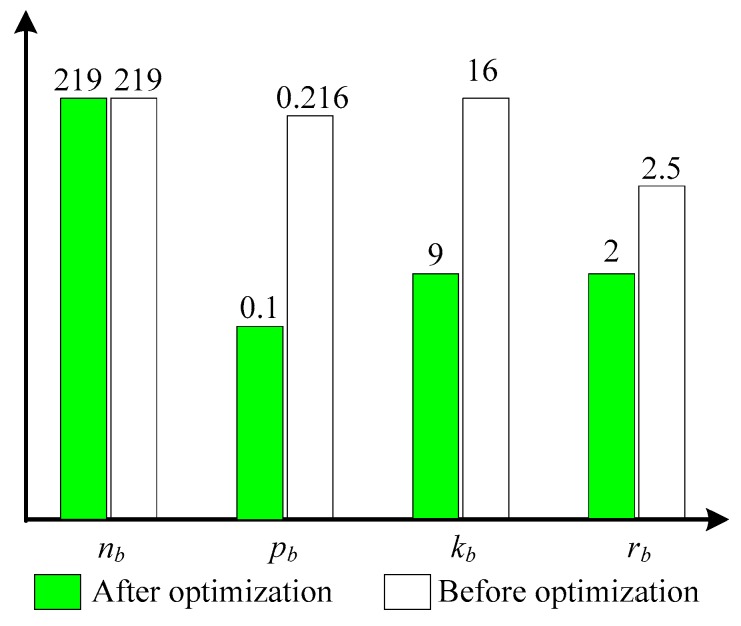
Part networking parameters -before and after optimization.

In the second round, we set *f*(*m*) = 2, namely, the second-smallest ratio of the communication radius, as detection radius. The optimization results are shown in line 3 of Table 5. The results of *c_b_*, *k_b_* are still decimals and the optimization results are not accurate. However, after disseminating the communication radius and detection radius information to the network, exact values *c_b_* = 1, *k_b_* = 9 can be obtained. At this point, the systematic performance values actually meet the requirement, so it reaches the final state. Comparing the situation before and after optimization, the change of some networking parameters is shown as Figure 11.

The experimental results show that by adjusting the networking parameters in our approach, the network systematic performance can achieve the mission objectives at minimal cost. Before deploying and running a network, the network can be optimized at any time according to the actual situation to change the performance of different dimensions, which provides a guarantee for the execution of actual missions.

## 7. Conclusions

Against the background that increasing attention is being paid to effectively safeguarding national marine rights and interests, the upsurge of marine economic development and significant progress in wireless sensor networks, underwater wireless sensor networks (UWSNs) have become a new hot research area. Nevertheless, due to the constantly changing network topology, and the fact that working times and scope of coverage are affected by the energy consumption, high variability in the acoustic channel, and the complex relationship between influencing factors and performance parameters, node deployment, networking, and performance calculation of UWSNs are challenging issues. This paper is centered on this topic and its major contribution lies in three aspects: (1) we have improved the Normal Body-centered Cubic Lattice to a Cross Body-centered Cubic Lattice (CBCL), and a Cross Deployment Networking Method (CDNM) of UWSNs suitable for the underwater environment is proposed too; (2) a Systematic Quar-Performance Calculation Model (SQPCM) is proposed, then the measurement models of systematic performance are established; (3) a Networking Parameters Adjustment Method (NPAM) for the optimized systematic performance of UWSNs is presented. Finally, two simulation experiments have been carried out, and the results demonstrate that the approach proposed by this paper is highly feasible and efficient in evaluating the systematic performance of UWSNs and optimizing UWSNs.

In the future, we plan to study the method of UWSN data storage and acquisition after achieving network deployment, networking and system performance analysis. For the data storage and acquisition of UWSN, we will establish a metadata model to describe the abstract form of data content and storage location, and build a center ring structure that is composed of nodes according to the shortest of average data query path, and the metadata is stored in the center ring nodes. When there is data that will be stored in of any nodes, the data is transmitted to the storage node and the metadata generated for it, then metadata is diffused and synchronized to the center ring nodes. When there is query data of any node by a user, the nearest center ring node will process the query sentence according the metadata, and the specific data will be sent to the user.

## Figures and Tables

**Table 1:** Parameters and labels.

Sensor Node Parameters	Label	Master and Mobile Node Parameters	Label
underwater communication radius	*r_b_*	mobile node velocity	*v_u_*
underwater communication bandwidth	*b_b_*	mobile node underwater communication bandwidth	*b_u_*
overwater communication bandwidth	*b’_b_*	mobile node detected radius	*d_u_*
detected radius	*d_b_*	mobile node detected precision	*h_u_*
detected precision	*h_b_*	mobile node number	*n_u_*
number	*n_b_*	mobile node total energy	*e_u_*
total energy	*e_b_*	mobile node energy consumption	*p_u_*
average energy consumption	*p_b_*	master node velocity	*v_s_*
average connectivity	*k_b_*	master node underwater communication bandwidth	*b_s_*
average coverage	*c_b_*	master node number	*n_s_*

**Table 2:** Parameter categories in the Systematic Performance Measurement Model.

Systematic Performance	Constraint Parameters	Device Performance Parameters	Networking Parameters
$\Theta =\alpha \cdot \frac{n_{b}\cdot \pi \cdot d_{b}^{3}}{c_{b}}\cdot \left[1-{\left(1-h_{b}\right)}^{c_{b}}\right]+\beta \cdot n_{u}\cdot d_{u}^{3}\cdot h_{u}$			
Coverage	*α*, *β*, *γ*	*h_b_*, *n_u_*, *d_u_*,*h_u_*	*d_b_*, *n_b_*, *c_b_*
$\Phi =\alpha \cdot k_{b}\cdot \left[I\cdot \omega \cdot \left(1-\eta \right)\cdot b_{b}+\left(1-\omega \right)\cdot b_{b}^{\prime }\right]+\beta \cdot I\cdot v_{u}\cdot n_{u}\cdot b_{u}$			
Connectivity	*α*, *ω*, *β*	*v_s_*, *n_s_*, *b_s_*, *b_b_*, *b_b_’*, *b_u_*, *v_u_*, *n_u_*	*k_b_*
$\Psi =\alpha \cdot \left(\underset{m}{\mathrm{Min}}\left(\frac{e_{b}}{p_{b}}\right)+\beta \cdot \frac{e_{u}}{p_{u}}\right)$			
Durability	*α*, *β*, *m*	*e_u_*, *e_b_*, *p_u_*	*p_b_*
$\Omega =\frac{\alpha \cdot k_{b}\cdot b_{b}}{\frac{L}{V}\cdot \left(1+\frac{s\cdot l}{\varphi }\right)}$			
Rapid-reactivity	*α*, *s*, *l*	*b_b_*	*r_b_*, *k_b_*, *c_b_*

**Table 3:** Durability comparison between NBCL and CBCL of UWSN.

	*r_b_* = 3.46	*r_b_* = 4	*r_b_* = 5.66	*r_b_* = 6.93
NBCL	1011.46 h ≈ 42 days	872.74 h ≈ 36 days	755.66 h ≈ 31 days	688.73 h ≈ 29 days
CBCL	1585.44 h ≈ 66 days	1129.4 h ≈ 51 days	1076.16 h ≈ 45 days	946.85 h ≈ 39.5 days

**Table 4:** SQPCM compared with some other works.

Item	Our work	Work [19]	Work [20]	Work [21]	Work [22]	Work [26]	Work [27]
Coverage	√	√	√	--	--	√	--
Connectivity	√	√	--	--	--	√	√
Durability	√	--	--	√	√	--	√
Rapid-reactivity	√	--	√	√	√	--	√

**Table 5:** Optimized networking parameters of UWSN.

	*n_b_*	*p_b_*	*d_b_*	*c_b_*	*k_b_*	*r_b_*
**First round**	219	0.088974	1000	1.1237	7.559	1903.5
**Second round**	219	0.099964	1000	1.1418	7.5763	2000.8
**Final state**	219	0.1	1000	1	9	2000
